# Efficacy of Methotrexate and Anti-TNF Combination Therapy in Adults with Refractory Crohn’s Disease

**DOI:** 10.34172/mejdd.2024.395

**Published:** 2024-10-30

**Authors:** Paria Boustani, Anahita Sadeghi, Sina Khayatian, Sudabeh Alatab, Amir Anushiravani, Ali Reza Sima, Homayoon Vahedi

**Affiliations:** ^1^Digestive Disease Research Center, Digestive Disease Research Institute, Tehran University of Medical Sciences, Tehran, Iran; ^2^Department of Internal Medicine, Tehran University of Medical Sciences, Tehran, Iran

**Keywords:** Crohn’s disease, Methotrexate, Anti-TNF, Inflammatory bowel disease

## Abstract

**Background:**

Biological medications have played a significant role in maintenance therapy for Crohn’s disease (CD), but some cases become refractory to these agents. Methotrexate (MTX) appears to be a cost-effective and readily available drug for enhancing the effectiveness of maintenance therapy when used in combination with anti-tumor necrosis factor (anti-TNF) therapy in such cases. However, its effectiveness is still to be established. We aimed to assess the efficacy of MTX and anti-TNF combination therapy in patients with refractory CD.

**Methods:**

A retrospective cohort study was conducted on adult patients with CD who were refractory to anti-TNF therapy and were initiated on weekly intravenous MTX in addition to the anti-TNF therapy. These patients were then followed up for over a year. The primary outcome measured was the clinical response to treatment, based on the Harvey-Bradshaw Index. The secondary outcomes included assessing the adverse events and complications of MTX therapy.

**Results:**

Of 70 patients, 44 were included in the final analysis. Among them, 30 patients (68.2%) achieved complete remission, four patients (9.1%) had a partial clinical response, and 10 patients (22.7%) required surgery. The adverse events and complications of MTX therapy were mild and infrequent (9.1%). None of the demographic or clinical factors were significantly associated with response to treatment (*P*>0.05).

**Conclusion:**

Combining MTX with anti-TNF therapy appears to be an effective and safe treatment for patients with Crohn’s disease, particularly those with severe disease who are less responsive to monotherapy. However, further studies are needed to confirm these findings.

## Introduction

 Crohn’s disease (CD) is becoming more prevalent worldwide, including in our country. Therefore, it is important to seek alternative maintenance therapy in cases where the standard treatments are not effective.^1,2^ Biological medications have played a significant role in maintenance therapy for moderate to severe CD, but some cases become resistant to these drugs due to antibody formation. In such instances, medications to reduce immunogenicity should be considered.^3^ The efficacy of methotrexate (MTX) in the treatment of steroid-dependent CD has been a debatable topic in the last two decades.^4-7^ MTX seems like a cost-effective and available potential alternative. Some studies have found MTX to be effective for maintenance therapy, while others have shown no clear advantage over other medications.^8-11^ Combining MTX with anti-tumor necrosis factor (anti-TNF) medication has been shown to enhance long-term maintenance therapy in patients with rheumatoid arthritis. This has prompted an evaluation of its immunomodulatory role in CD as well.^12-15^ Due to insufficient data about MTX therapy in CD in our country, we conducted a study to assess the efficacy of MTX and anti-TNF combination therapy in patients with refractory CD.

## Materials and Methods

###  Study Design and Participants 

 This was a retrospective cohort study conducted at two referral centers in Tehran, Iran (Shariati Hospital’s Gastroenterology Clinic and Masoud Clinic) from 2018 to 2023. The study population consisted of adult patients with CD who were refractory to anti-TNF monotherapy. These patients were initiated on a 25 mg weekly intravenous MTX regimen in addition to anti-TNF therapy, followed by a maintenance dosage of 15 mg weekly after 2 months. The anti-TNF agents used in the study were biosimilar adalimumab (CinnoRA® produced by CinnaGen CO.) and infliximab (REMICADE®). Inclusion criteria encompassed patients with confirmed CD diagnosed via endoscopy and histology, active disease defined by a Harvey Bradshaw Index (HBI) of more than 5, previous failure or loss of response to anti-TNF monotherapy, initiation of MTX and anti-TNF combination therapy, and a follow-up duration of over a year. Exclusion criteria were renal insufficiency (GFR < 60) or impaired liver enzymes, discontinuation of MTX due to pregnancy or complications, and missing follow-up data. The final analysis comprised 44 patients who met the inclusion and exclusion criteria, utilizing an intention-to-treat (ITT) approach.

###  Data Collection

 The patients’ data were obtained from medical records, including demographic information (age, sex), medical history (disease duration, previous treatments), CD type (location, behavior), laboratory data (complete blood count [CBC], erythrocyte sedimentation rate [ESR], C-reactive protein [CRP], fecal calprotectin [FC], liver enzymes), HBI scores at baseline and follow-up, adverse events and MTX therapy complications, and the necessity for surgery post-therapy

###  Outcome Measures

 The primary outcome was the clinical response to treatment, which was classified as complete remission (HBI < 5), partial response (HBI reduction of 3 or more from baseline), or treatment failure (HBI reduction of less than 3 from baseline or need for surgery). The secondary outcomes were the adverse events and complications of MTX therapy.

###  Statistical Analysis

 The data were analyzed using SPSS software version 27. Descriptive statistics were used to summarize the characteristics of the patients and the outcomes of the treatment. Inferential statistics were used to compare the outcomes between different groups of patients based on their demographic and clinical factors. The chi-square test or Fisher’s exact test was used for categorical variables, and *t* test or Mann-Whitney U test was used for continuous variables. A *P* value of less than 0.05 was considered statistically significant.

## Results

**Figure 1:**
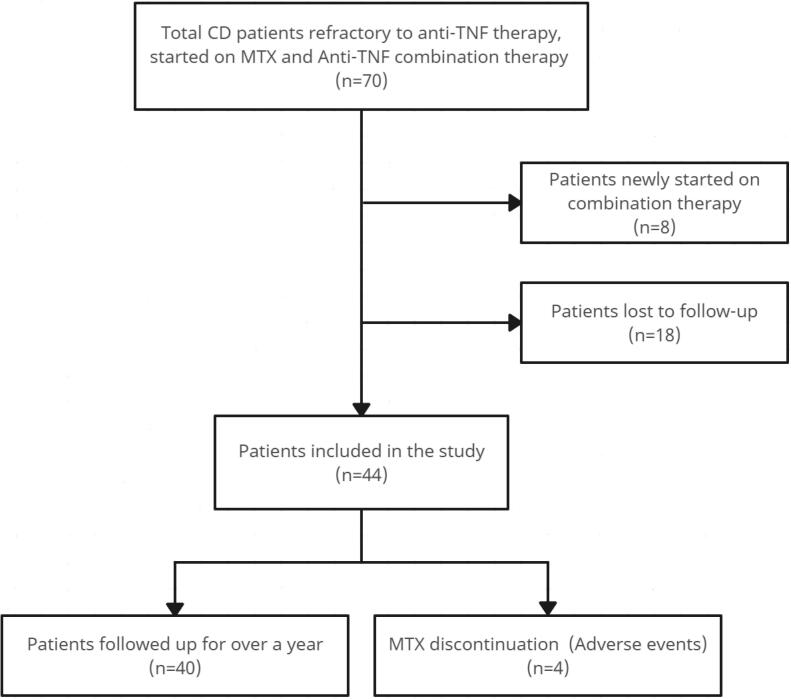
Patient selection flowchart

 The purpose of this study was to evaluate the effectiveness of MTX and anti-TNF combination therapy in patients with CD who were refractory to anti-TNF monotherapy. Out of 70 patients who began combination therapy, 44 were included in the final analysis (Figure 1). The mean age of the participants was 35 ± 10 years, and 59.1% were male. The baseline characteristics of patients are shown in Table 1. The primary outcome was the clinical response to treatment, which was categorized as complete remission, partial response, or treatment failure. The secondary outcomes were the adverse events and complications of MTX therapy. The results showed that 30 patients (68.2%) achieved complete remission, four patients (9.1%) had a partial response, and 10 patients (22.7%) required surgery. None of the demographic or clinical factors were significantly associated with the clinical response (*P* > 0.05). The adverse events and complications of MTX therapy were mild and infrequent. Only four patients (9.1%) discontinued MTX due to nausea, vomiting, or elevated liver enzymes. The details of the adverse events and the outcomes of the patients who discontinued MTX are presented in Table 2.

**Table 1:** Baseline characteristics of patients

**Characteristic**	**Total patients (n=44) **
Male, No. (%)	26 (59.1)
Female, No. (%)	18 (40.9)
Age at start of MTX (years), Mean (SD)	35 (10)
Disease duration (years), Mean (SD)	8 (6.4)
Location of disease, No. (%)	
Ileocolonic	22 (50)
Colonic	19 (43.2)
Ileal	3 (6.8)
Behavior of disease, No. (%)	
Fistulizing	24 (54.5)
Stricturing	13 (29.5)
Non-fistulizing & Non-stricturing	11 (25)
Type of anti-TNF agent, No. (%)	
Infliximab	15 (34.1)
Biosimilar adalimumab	29 (65.9)

**Table 2:** Adverse events in patients with CD receiving MTX

**MTX discontinuation**	**n (%)**	**Follow up**
Adverse events	4 (9.1)	
GI disorder	3 (6.82)	
Nausea & vomiting		-Started on biosimilar adalimumab + azathioprine, partial clinical response
Nausea & vomiting		-Started on infliximab + azathioprine, partial clinical response
Nausea & vomiting		-Required surgery
Impaired liver enzymes	1 (2.27)	-Moved to Canada and was started on ustekinumab, complete clinical response

## Discussion

 The main finding of the current study was that combination therapy resulted in a high rate of remission (68.2%) and a low rate of treatment failure (22.7%), compared with previous studies using MTX or anti-TNF monotherapy.^10,12^ This finding supports the hypothesis that MTX enhances the efficacy of anti-TNF agents by reducing their immunogenicity and preventing antibody formation.^4^ It also confirms the role of MTX as an immune modulator and a maintenance therapy in patients with CD.^11,16-20^

 Our finding is consistent with a small study by Schröder et al^3^, which showed a higher remission rate with infliximab (IFX) and MTX combination therapy (71%) than with IFX monotherapy (33.3%). However, our finding contrasts with a larger study by Feagan et al,^21^ which found no significant difference between IFX and MTX combination therapy and IFX monotherapy in terms of treatment failure rate (30.6% vs 29.8%). A possible explanation for this discrepancy is the difference in the study populations: Feagan et al^21^ included patients with moderate to severe CD, while our study included mostly patients with severe CD (75%). Therefore, combination therapy may be more beneficial for patients with more advanced disease who are less responsive to monotherapy.

 Our study showed a high tolerability of MTX among patients (90.9%), in contrast with previous studies where one-third of patients were intolerant.^22^ Only three (6.82%) cases experienced nausea and vomiting, and only one case (2.27%) had elevated liver enzymes as complications of MTX therapy. This indicates that MTX is a safe choice for patients with CD when used in combination with anti-TNF agents.

 A major limitation of our study was the small sample size, which may have reduced the statistical power and the representativeness of the results. Moreover, the lack of a control group receiving MTX or anti-TNF monotherapy prevented us from directly comparing the efficacy of combination therapy with either agent alone. Therefore, our results may not be generalizable to other populations and settings, and causal relationships cannot be established. Future studies should use larger and more diverse samples, include control groups with different treatments, and employ more rigorous methods to reduce selection bias and increase validity.

## Conclusion

 In conclusion, our study showed that MTX and anti-TNF combination therapy is an effective and safe treatment for patients with CD who are refractory to monotherapy. Further studies are needed to confirm these findings and to explore the optimal dose, duration, and timing of combination therapy.

## References

[1] Ng SC, Shi HY, Hamidi N, Underwood FE, Tang W, Benchimol EI (2017). Worldwide incidence and prevalence of inflammatory bowel disease in the 21st century: a systematic review of population-based studies. Lancet.

[2] Safarpour AR, Hosseini SV, Mehrabani D (2013). Epidemiology of inflammatory bowel diseases in Iran and Asia; a mini review. Iran J Med Sci.

[3] Cushing K, Higgins PD (2021). Management of Crohn disease: a review. JAMA.

[4] Herfarth HH (2016). Methotrexate for inflammatory bowel diseases - new developments. Dig Dis.

[5] Rosh JR (2020). The current role of methotrexate in patients with inflammatory bowel disease. Gastroenterol Hepatol (N Y).

[6] Sun JH, Das KM (2005). Low-dose oral methotrexate for maintaining Crohn’s disease remission: where we stand. J Clin Gastroenterol.

[7] Colman RJ, Lawton RC, Dubinsky MC, Rubin DT (2018). Methotrexate for the treatment of pediatric Crohn’s disease: a systematic review and meta-analysis. Inflamm Bowel Dis.

[8] Patel V, Macdonald JK, McDonald JW, Chande N. Methotrexate for maintenance of remission in Crohn’s disease. Cochrane Database Syst Rev 2009(4):CD006884. 10.1002/14651858.CD006884.pub2. 19821390

[9] Alfadhli AA, McDonald JW, Feagan BG. Methotrexate for induction of remission in refractory Crohn’s disease. Cochrane Database Syst Rev 2005(1):CD003459. 10.1002/14651858.CD003459.pub2. 15674908

[10] Feagan BG, Rochon J, Fedorak RN, Irvine EJ, Wild G, Sutherland L (1995). Methotrexate for the treatment of Crohn’s disease The North American Crohn’s Study Group Investigators. N Engl J Med.

[11] Cassinotti A, Batticciotto A, Parravicini M, Lombardo M, Radice P, Cortelezzi CC (2022). Evidence-based efficacy of methotrexate in adult Crohn’s disease in different intestinal and extraintestinal indications. Therap Adv Gastroenterol.

[12] Breedveld FC, Weisman MH, Kavanaugh AF, Cohen SB, Pavelka K, van Vollenhoven R (2006). The PREMIER study: a multicenter, randomized, double-blind clinical trial of combination therapy with adalimumab plus methotrexate versus methotrexate alone or adalimumab alone in patients with early, aggressive rheumatoid arthritis who had not had previous methotrexate treatment. Arthritis Rheum.

[13] Schröder O, Blumenstein I, Stein J (2006). Combining infliximab with methotrexate for the induction and maintenance of remission in refractory Crohn’s disease: a controlled pilot study. Eur J Gastroenterol Hepatol.

[14] Kotsiliti E (2023). Combination therapy with methotrexate in paediatric Crohn’s disease. Nat Rev Gastroenterol Hepatol.

[15] Sokol H, Seksik P, Carrat F, Nion-Larmurier I, Vienne A, Beaugerie L (2010). Usefulness of co-treatment with immunomodulators in patients with inflammatory bowel disease treated with scheduled infliximab maintenance therapy. Gut.

[16] Wang M, Zhao J, Wang H, Zheng C, Chang B, Sang L (2022). Methotrexate showed efficacy both in Crohn’s disease and ulcerative colitis, predictors of surgery were identified in patients initially treated with methotrexate monotherapy. Front Pharmacol.

[17] Feagan BG, Fedorak RN, Irvine EJ, Wild G, Sutherland L, Steinhart AH (2000). A comparison of methotrexate with placebo for the maintenance of remission in Crohn’s disease North American Crohn’s Study Group Investigators. N Engl J Med.

[18] Nielsen OH, Steenholdt C, Juhl CB, Rogler G (2020). Efficacy and safety of methotrexate in the management of inflammatory bowel disease: a systematic review and meta-analysis of randomized, controlled trials. EClinicalMedicine.

[19] Patel V, Wang Y, MacDonald JK, McDonald JW, Chande N (2014). Methotrexate for maintenance of remission in Crohn’s disease. Cochrane Database Syst Rev.

[20] Lémann M, Zenjari T, Bouhnik Y, Cosnes J, Mesnard B, Rambaud JC (2000). Methotrexate in Crohn’s disease: long-term efficacy and toxicity. Am J Gastroenterol.

[21] Feagan BG, McDonald JW, Panaccione R, Enns RA, Bernstein CN, Ponich TP, et al. Methotrexate in combination with infliximab is no more effective than infliximab alone in patients with Crohn’s disease. Gastroenterology 2014;146(3):681-8.e1. 10.1053/j.gastro.2013.11.024. 24269926

[22] Hong HS, Kim K, Oh K, Lee JY, Hong SW, Park JH (2021). Short-term tolerability and effectiveness of methotrexate monotherapy in adult patients with Crohn’s disease: a retrospective study. Therap Adv Gastroenterol.

